# School bullying and non-suicidal self-injury: the mediating role of depression and the moderating role of social support

**DOI:** 10.3389/fpsyg.2025.1557400

**Published:** 2025-05-30

**Authors:** Yongzhi Jiang, Haifei Chai

**Affiliations:** ^1^School of Educational Science, Inner Mongolia Minzu University, Tongliao, China; ^2^Inner Mongolia Autonomous Region Student Bullying Prevention and Control Research Center, Tongliao, China; ^3^Research Base for Educational and Psychological Development of Nationalities in Inner Mongolia, Tongliao, China

**Keywords:** single-parent families, school bullying, non-suicidal self-injury, depression, social support

## Abstract

**Objective:**

To explore the relationship between school bullying and non-suicidal self-injury and the roles of depression and social support among high school students from single-parent families.

**Methods:**

The School Bullying Questionnaire, the Adolescent Non-Suicidal Self-Injurious Behavior Rating Questionnaire, the Stream Call Depression Self-Rating Scale, and the Adolescent Social Support Scale were used to investigate 312 high school students from single-parent families.

**Results:**

(1) School bullying positively predicted non-suicidal self-injurious behaviors among high school students from single-parent families; (2) depression mediated the relationship between school bullying and non-suicidal self-injurious behaviors among high school students from single-parent families; and (3) social support negatively moderated the second half of the pathway and the direct pathway of this mediation model.

**Conclusion:**

There is a moderated mediating effect between school bullying and non-suicidal self-injury among high school students from single-parent families, depression is a mediating variable in the relationship, and social support mitigates the effects of school bullying and depression on non-suicidal self-injurious behavior.

## 1 Introduction

As the level of social development increases, the divorce rate rises year by year. According to the China Marriage and Family Report 2023 Edition China’s divorce rate rose from 0.96% in 2000 to 2% in 2010, to 3% in 2016, and to a peak of 3.4% in 2019, which led to an increasing number of single-parent families. In addition, because China is more focused on family culture and the importance of ethics and collectivity than other countries, the absence of family structure is more likely to lead to a reduction or even a shortage of family resources provided by the father’s generation to the offspring, and the pattern of intergenerational interactions is more likely to be distorted, which can in turn lead to the emergence of mental health problems. In particular, for the group of high school students from single-parent families, this stage of high school students’ self-consciousness is high, and their state of mind is more prone to change, and high school students in this sensitive period are more prone to mental health problems that can lead to self-injury and other behavioral problems. A meta-analysis based on 10 years of data from 2012 to 2022 showed that the total detection rate of non-suicidal self-injury among adolescents in a healthy population in China was 25% (Tao et al., 2023). Ren et al. (2024) study showed that non-suicidal self-injury among secondary school students was associated with parental divorce. The study showed that non-suicidal self-injury in secondary school students was associated with parental divorce. Non-suicidal self-injury (NSSI) behaviors is generally defined as an intentional act of injury to one’s own bodily tissues that is not aimed at suicide. This behavior, while generally not life-threatening, has a greater negative impact in both the short and long term. A follow-up study showed that adolescents with stable repetitive NSSI behaviors exhibited significantly higher levels of stress and anxiety 10 years later (Daukantaitė et al., 2021). Furthermore, a study by Ruan et al. (2022) showed that high frequency of self-injury in adolescents is associated with suicidal ideation. Existing studies have focused on the influencing factors, coping styles, and intervention strategies of NSSI behaviors, but there are fewer studies on NSSI behaviors of the special group of high school students from single-parent families. This special group was chosen as the target of this study, aiming to reduce the occurrence and negative effects of NSSI behavior through the study of its intrinsic mechanisms.

NSSI behavior involves numerous influences, and a large number of studies have shown that school bullying is a positive predictor of adolescent NSSI behavior. School bullying refers to a situation within a school setting in which a more powerful party intentionally harms a weaker party and causes harm to the weaker party (Zhang, 2023). The behavior in which the stronger party intentionally harms the weaker party and will bring harm to the weaker party. School bullying, as a vicious incident in school, is likely to lead to physical and mental problems such as anxiety, depression, and self-injury among students (Feng et al., 2024; Bu and Lu, 2024). Some studies have found that there is a positive correlation between being bullied by peers and self-injurious behaviors such as high and low fatal self-injurious behaviors and potential injuries (Ye et al., 2023). The Kennedy and Brausch (2024) study also found that adolescent bullying exposure was associated with an increased risk of suicide attempts and NSSIs. All of the aforementioned studies found that adolescents who were exposed to bullying were at a higher risk for potential self-injury. In light of this, this study proposes Hypothesis 1 (H1): School bullying positively predicts non-suicidal self-injurious behavior in high school students from single-parent families.

A meta-analysis of the correlation between depression and NSSI showed a correlation between the two (Li et al., 2016). Also, according to the experiential avoidance model of self-injury, individuals who want to avoid or alleviate the negative effects produced by a person’s stimulus will likely engage in self-injurious behavior (Xiang et al., 2019). This behavior results in negative reinforcement while avoiding or alleviating the negative impact, and when the individual encounters the negative stimulus again, he or she will adopt non-suicidal self-injurious behavior, forming an automated escape response. The result of this behavior is a negative reinforcing effect (Chapman et al., 2006). Therefore, depression, as a negative emotion, is highly likely to induce non-suicidal self-injurious behavior. Previous studies have shown that school bullying tends to lead to depression in students and that depression predicts the occurrence of non-suicidal self-injurious behavior and depression can predict the occurrence of non-suicidal self-injurious behaviors (Hong et al., 2024; Yin et al., 2024). Therefore, this study proposes Hypothesis 2 (H2): Depression mediates school bullying and NSSI self-injurious behaviors among high school students from single-parent families.

However, not all high school students from single-parent families with high levels of school bullying and depression develop non-suicidal self-injurious behaviors. A study by Guo and Wu (2023) showed that increased social support was effective in reducing the incidence of NSSI behaviors. Social support is the help an individual receives from the outside world, including material and spiritual support (Yuan et al., 2023). According to social support theory, social support networks help individuals cope with various external challenges. In other words, when an individual is bullied or develops depressive symptoms, the presence of social support can provide the individual with the energy to cope with the challenges and avoid the development of further negative behaviors. Huang et al.’s (2023) study showed that higher levels of social support were effective in reducing the risk of NSSI behaviors among depressed medical students. A study by Claes et al. (2015) showed that the relationship between bullying and NSSI and depressed mood and NSSI was found to be moderated by parental support. Accordingly: this study proposes Hypothesis 3 (H3): Social support moderates the direct path and the second half of the indirect path of this mediation model.

**Figure 1:**
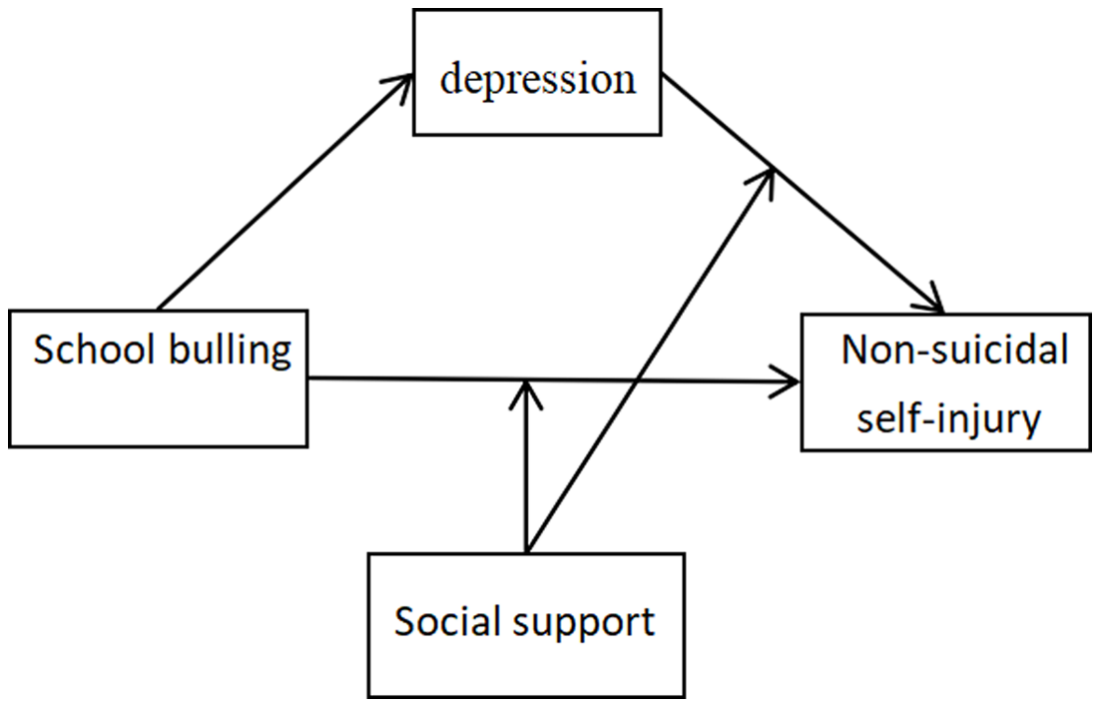
The hypothetical model underlying the study.

In summary, the hypothetical model for this study is shown in Figure 1. Current research has not yet fully explored the internal mechanisms of bullying victimization and NSSI behavior. And less attention has been paid to the special group of high school students from single-parent families. Therefore, the present study intends to explore the effects of school bullying on NSSI behaviors among high school students from single-parent families and its mechanisms of action, focusing on the mechanisms of depression and social support, with a view to providing interventions for NSSI behaviors among children from single-parent families.

## 2 Research methodology

### 2.1 Study subjects

A convenience sampling method was used to select 1,300 high school students from three high schools in Jiangsu Province, and 312 high school students from single-parent families were screened out on the basis of their family structure as “single-parent” (including divorced and widowed), with an average age of 16.35 ± 0.58 years old; among them, 132 were boys and 180 were girls; 186 were in senior high school; 126 were in senior high school; 148 were from urban and 164 were from rural populations. 126; 148 urban and 164 rural.

### 2.2 Research tools

#### 2.2.1 School bullying questionnaire

The Bullying Scale from the Olweus Bullying Questionnaire, revised version of Zhang et al. was used. The questionnaire was administered retrospectively over a three-month period and asked about the number of times the subjects had been bullied by their classmates at school. The questionnaire consisted of 6 questions and was scored on a 5-point Likert scale, with 5 indicating “several times a week” and 1 indicating “none.” The total score positively indicates the degree of bullying of an individual. The Cronbach’s alpha coefficient for the scale in this study was 0.64, the KMO value of 0.70.

#### 2.2.2 Questionnaire for rating non-suicidal self-injurious behavior in adolescents

The Adolescent Non-Suicidal Self-Injury Rating Questionnaire developed by Wan Yuhui et al. was used, which contains two dimensions with 12 questions, and is scored on a five-point Likert scale, i.e., “no~always,” on a scale of 0–4, respectively, with the score being positively indicative of the severity of the adolescent’s NSSI behavior. The Cronbach’s *α* of the questionnaire in this study was 0.81, the KMO value of 0.79.

#### 2.2.3 Streaming call depression self-rating scale

The Center for Epidemiological Studies Depression Scale developed by Radloff was used, which consists of 20 items based on occurrences in the past week, with less than one day as “none or almost none” and 5–7 days as “almost all the time,” and rated in the order of 3, 2, 1, and 0, with 4, 8, 12, and 16 being reverse scored. The Cronbach’s alpha coefficient for this study was 0.90, the KMO value of 0.91.

#### 2.2.4 Adolescent social support scale

The Social Support Scale for Youth and Young Adults (SSSYA) compiled by Yeh et al. was used, with a total of 17 questions and three dimensions. A five-point Likert scale was used, with 1 indicating “does not meet” and 5 indicating “meets,” and a positive score indicating the level of social support. The Cronbach’s alpha coefficient for the scale in this study was 0.92, the KMO value of 0.91.

### 2.3 Measurement procedures

The survey was conducted anonymously and the questionnaires were distributed in the classroom as a class and collected on the spot. Before the test, the subjects were informed of the purpose of the questionnaire, that there was no right or wrong answer, and that all information would be kept strictly confidential for academic research purposes only, and that submission of the questionnaire would be considered as informed consent, which was obtained from the class teacher and the subjects. The consent of the class teacher and the subjects was obtained before the test. The main test was conducted by the mental health teacher, who explained the requirements for answering the questionnaire and the issues to be noted, and informed the students that they could ask questions at any time if they had any doubts during the process of filling out the questionnaire. Group counseling with a social support theme will be conducted as a class for groups reporting high levels of distress on the survey and reported to the mental health teacher for further attention.

### 2.4 Data analysis

SPSS27.0 and Process4.1 macro program were used for data processing and analysis.

## 3 Findings

### 3.1 Common method bias test

Exploratory factor analysis was performed using the Harman one-factor test. All question items included in the four variables of school bullying, non-suicidal self-injury, depression, and social support were included in the exploratory factor analysis. The results showed that there were 14 factors with an eigenroot greater than 1. The first factor explained 21.77% of the total variance, which is less than the critical value of 40%, indicating that there was no significant common method bias in this study.

### 3.2 Descriptive statistics and correlation analysis of variables

Table 1 shows that school bullying, depression, social support and non-suicidal self-injury were both correlated (*p* < 0.05), in which school bullying was significantly positively correlated with depression and non-suicidal self-injury, respectively (*p* < 0.01), and significantly negatively correlated with social support (*p* < 0.05); social support was significantly negatively correlated with depression and non-suicidal self-injury, respectively (*p* < 0.05); depression was significantly positively correlated with non-suicidal self-injury (*p* < 0.01).

**Table 1:** Descriptive statistics and correlation analysis of variables.

Variable	*M*	SD	Bullying in schools	Depression	Social support	Non-suicidal self-injury
Bullying in schools	8.17	3.01	1			
Depression	22.77	10.70	0.24^**^	1		
social support	59.66	14.78	−0.11^*^	−0.44^**^	1	
Non-suicidal self-injury	3.49	5.04	0.20^**^	0.47^**^	−0.32^**^	1

*P < 0.05, **P < 0.01, ***P < 0.001.

### 3.3 Mediated effects test

The mediating effect of depression and the moderating effect of social support were tested sequentially. Data were first standardized, controlling for gender and grade level, and secondly, Model 4 of the SPSS macro program PROCESS was used to test the mediating effect of depression. As shown in Table 2, school bullying was an extremely significant positive predictor of depression (*β* = 0.87, *p* < 0.001), and the fit index *R*^2^ showed that the predictive model of school bullying on depression explained 6% of its variance. When both school bullying and depression predicted non-suicidal self-injurious behavior, non-suicidal self-injurious behavior could not be significantly predicted by school bullying (*β* = 0.16, *p* > 0.05), meanwhile, depression was a significant predictor of NSSI behavior (*β* = 0.21, *p* < 0.001).

**Table 2:** Model test of the mediating role of depression as a mediating variable in school bullying on non-suicidal self-injurious behavior.

Outcome variable	Predictor variable	*b*	SE	*T*	95% Confidence interval		*R* ^2^	*F*
					LLCI	ULCI		
Depression	Bullying in schools	0.87	0.20	4.43^***^	0.48	1.26	0.06	19.65
Non-suicidal self-injury	Bullying in schools	0.16	0.09	1.84	−0.01	0.33	0.23	45.98
	Depression	0.21	0.02	8.68^***^	0.16	0.26		

As shown in Table 3, depression mediates the association between school bullying and non-suicidal self-injurious behavior among high school students from single-parent families. Specifically, at the 95% confidence interval, the results of the mediation test included 0 for the direct effect (LLCI = −0.01, ULCI = 0.33, *p* > 0.05), and did not include 0 for the indirect effect (LLCI = 0.09, ULCI = 0.30, *p* < 0.001), which resulted in a significant mediating role for depression in the mediation between school bullying and NSSI behavior.

**Table 3:** Analysis of the mediating effect of depression in school bullying on NSSI behavior.

Effect type	Efficiency value	SE	BootLLCI	BootULCI	Relative effect value
Aggregate effect	0.34	0.09	0.16	0.53	
Direct effect	0.16	0.09	−0.01	0.33	
Indirect effect	0.18	0.05	0.09	0.30	100%

### 3.4 Moderating effects test

Model 59 in PROCESS 4.1 modeling was used to test the mediation model with moderation after controlling for gender. The results, as shown in Table 4, showed that the interaction terms of social support with school bullying and its interaction with depression were significant predictors of NSSI behavior (*β* = 0.02, *p* < 0.01; *β* = −0.01, *p* < 0.001), and that its interaction with school bullying was not significant predictor of depression (*β* = −0.01, *p* > 0.05). This suggests that social support moderates the direct path and the second half of the path of this mediation model.

**Table 4:** Mediation effects test with moderation.

Outcome variable	Predictor variable	Overall fit index		Significance of regression coefficients		
		*R* ^2^	*F*	*β*	SE	*t*
Depression	Gender	0.29	30.78***	5.05	1.06	4.78***
	Bullying in schools			1.11	0.63	1.77
	Social support			−0.26	0.10	−2.69**
	School bullying × social support			−0.01	0.01	−0.56
Non-suicidal self-injury	Gender	0.30	21.41***	0.97	0.51	1.89
	Bullying in schools			−0.82	0.31	−2.68**
	Depression			0.51	0.10	5.18***
	Social support			0.05	0.08	0.66
	School bullying × social support			0.02	0.01	3.30**
	Depression × social support			−0.01	0.00	−3.55***

**Figure 2:**
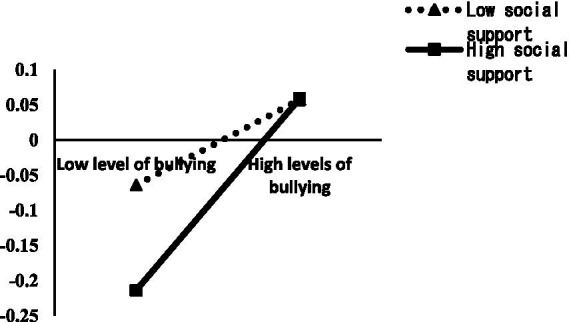
Moderating effect of social support between school bullying and non-suicidal self-injury.

**Figure 3:**
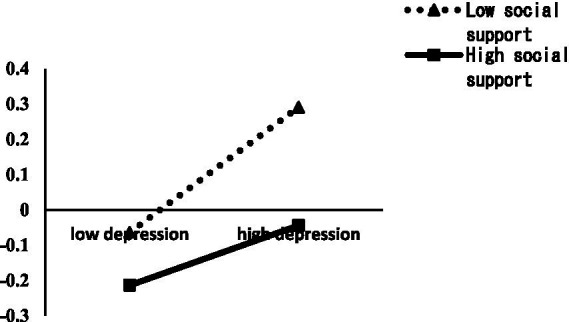
Moderating effects of social support between depression and non-suicidal self-injury.

In order to more clearly show the moderating effect of social support, the scores of social support were divided into two groups of high and low by plus or minus 1 standard deviation, and simple slope analysis was used to further examine the moderating effect of social support between bullying and non-suicidal self-injury (NSSI) at school, and the moderating effect of social support between depression and NSSI. As shown in Figure 2, bullying had a greater positive predictive effect on NSSI behavior at lower levels of social support (bsimple = 0.13, *p* < 0.05), and the predictive effect of bullying on NSSI behavior was significantly weaker at higher levels of social support (bsimple = 0.28, *p* < 0.001). This indicates that the predictive effect of bullying on NSSI behavior tends to weaken with higher levels of social support. As shown in Figure 3, depression had a greater positive predictive effect on NSSI behavior at lower levels of lower social support (bsimple = 0.35, *p* < 0.001), and depression had a significantly weaker predictive effect on NSSI behavior at lower levels of social support (bsimple = 0.17, *p* < 0.05). This indicates that the predictive effect of depression on NSSI behavior tends to weaken with increasing levels of social support.

## 4 Discussion

The results of this study indicate that school bullying significantly and positively predicts NSSI behaviors among high school students from single-parent families, consistent with Hypothesis 1. This result is consistent with previous research (Zhang et al., 2024). It is also consistent with the experiential avoidance model of self-injury, in which students who suffer from school bullying may develop negative emotions such as shame and fear, and choose self-injurious behaviors to avoid them when there is no proper way to detach them. In addition, the interpersonal/systemic model of self-injury suggests that Non-suicidal self-injury can help individuals promote help-seeking behavior (Ye et al., 2023). After bullying victimization, help-seeking families are less cohesive and intimate than two-parent families on the family side, and students on the school side are worried about increased bullying, thus releasing emotions through self-injurious behaviors. Thus, school bullying significantly and positively predicted non-suicidal self-injurious behavior among high school students from single-parent families. This reveals the need for parents and schools to guard against school bullying, and even if it occurs, to take timely and appropriate measures to address it, provide comfort and support to the bullied, and avoid students’ self-injury.

This study also found that depression fully mediated between school bullying and non-suicidal self-injurious behavior among high school students from single-parent families, consistent with Hypothesis 2. Previous studies have shown that depression significantly mediates between cyber bullying and adolescent NSSI (Zhang et al., 2021). This study confirms the mediating role of depression in the traditional bullying domain and completes the research on the relationship between bullying and NSSI behavior. The same validates the experiential avoidance model of self-injury, which shows that the mechanism of self-injury formation is that an external stimulus triggers a negative emotion in the individual, and the individual commits self-injurious behaviors in order to avoid or alleviate the experience of this negative emotion in the context of the interplay of multiple factors. Depression, as a negative emotion, triggers self-injurious behavior through this mechanism. High school students from single-parent families who are in the unfavorable social relationship situation of bullying will increase the risk of depressed mood or depressive mental illness, which in turn will produce NSSI behaviors. This reveals that it is important to pay close attention to the mood of students after bullying and take timely interventions if depressive mood is detected to avoid further adverse behaviors.

The results of the present study showed that social support plays a moderating role in the mediation model of school bullying affecting self-injurious behaviors of high school students from single-parent families through depression, validating Hypothesis 3. First, individuals with high levels of social support had a higher predictive effect than those with low levels of social support. However, it is worth mentioning that the predictive roles of the two were similar at high bullying levels in this study. This suggests that the moderating effect of social support alone is not sufficient to lead to a reduction in self-injurious behavior when bullying levels are too high. This is consistent with the Dynamic Modal theory of social support (Dynamic Modal), i.e., the relationship between stress and social support, i.e., the relationship between stress and social support is bidirectional, interactive, and changes over time (Lü, 2010). Second, depression was a higher predictor of self-injurious behavior in individuals with low levels of social support than in individuals with high levels of social support, consistent with previous research findings. Consistent with the experiential avoidance model of self-injury, this model emphasizes that individuals who develop negative emotions produce self-injurious behaviors in response to the interaction of multiple factors, including high emotional intensity, difficulties in emotion regulation, and lack of emotion regulation strategies. Social support can provide individuals with good emotion regulation strategies and help them regulate their emotions, weakening the predictive role of depression on self-injurious behavior. It the same time, in a social structure like China’s, which is based on blood ties, collectivism and family are given great importance, so support from family can modulate the damage that depression and other negative emotions can cause to an individual. This result suggests that we need to enhance social support for high school students from single-parent families to enable them to adopt adaptive ways to cope with negative life events and reduce self-injurious behaviors. Building social networks through parental care, teacher attention and student care. That is, first, to enhance the parent–child relationship connection, such as Wenzhou City, Zhejiang Province, China, launched a volunteer service program for children’s visitation rights, which provides services to the party who does not directly support the children in a divorced family through the helpers, escorts, social workers and so on, so as to effectively increase the length of parent–child meetings and promote the establishment of social support networks for students from single-parent families. Second, pay special attention to students from single-parent families. Teachers can borrow tools, such as daily mood stickers, to understand students’ emotional changes, and for students who have expressed negative emotions for many times in a row, teachers can talk to them privately or seek help from psychologists. Third, enhance students’ mutual help and socialization. Through cooperative learning and labor, students from single-parent families can enrich their interpersonal communication pathways, give full play to their collective strength so that they can experience the joy of building close relationships and promote the construction of a social support system.

This study reveals the impact of school bullying on non-suicidal self-injurious behavior of high school students from single-parent families and its internal mechanism of action, which is a theoretical guide for the effective prevention and control of NSSI behavior of high school students from single-parent families. However, there are still some shortcomings. First, the sample size of this study is relatively small and the convenience sampling method may introduce selection bias. This may lead to the results being affected by outliers and random errors, and the generalization is limited. Future studies should expand the sample size and choose more scientific sampling methods, such as simple random sampling. Second, although the effects of gender and grade level on NSSI behavior were controlled for in the data analysis, the effects of socioeconomic status, parental education, and duration of single-parent status have not been considered. Future research should consider the effects of these factors on NSSI behavior among high school students from single-parent families to strengthen the validity of the article. Finally, this study was a cross-sectional study, limiting the ability to infer causality. In the future, there is a need to provide stronger evidence for the proposed mechanism more objectively and comprehensively using a tracer study approach.

## Data Availability

The original contributions presented in the study are included in the article/supplementary material, further inquiries can be directed to the corresponding author/s.
